# Fetal demise and associated factors following umbilical cord prolapse in Mulago hospital, Uganda: a retrospective study

**DOI:** 10.1186/1742-4755-11-12

**Published:** 2014-02-01

**Authors:** Esau Wangi Wasswa, Sarah Nakubulwa, Twaha Mutyaba

**Affiliations:** 1 Department of Obstetrics and Gynecology, Makerere University College of Health Sciences, PO Box 7072, Kampala, Uganda

**Keywords:** Fetal demise, Umbilical cord prolapse, Uganda

## Abstract

**Background:**

Umbilical cord prolapse is an obstetric complication associated with high perinatal morbidity and mortality. A few interventions may improve fetal outcome. In developed countries these have advanced to giving intrauterine fetal resuscitation. Conditions in low resource settings do not allow for some of these advanced techniques. Putting the mother in knee chest position and immediate delivery may be the only options possible.

We set out to determine the incidence of fetal demise and associated factors following umbilical cord prolapsed (UCP) in Mulago Hospital, Uganda.

**Methods:**

In a retrospective study conducted in Mulago hospital, Uganda, file records of mothers who delivered between 1^st^ January 2000 to 31^st^ December 2009 and had pregnancies complicated by umbilical cord prolapse with live fetus were selected. We collected information on referral status, cord position, cervical dilatation, fetal heart state at the time of diagnosis of UCP, diagnosis to delivery interval, use of knee chest position, mode of delivery, birth weight and fetal outcome.

We computed incidence of fetal demise following UCP and determined factors associated with fetal demise in pregnancies complicated by UCP.

**Results:**

Of 438 cases with prolapsed cord, 101(23%) lost their babies within 24 hours after birth or were delivered dead. This gave annual cumulative incidence of fetal death following UCP of 23/1000 live UCP cases delivered /year.

The major factors associated with fetal outcome in pregnancies complicated by UCP included; diagnosis to delivery interval <30 min, RR 0.79 (CI 0.74-0.85), mode of delivery, RR 1.14 (CI 1.02-1.28), knee chest position, RR 0.81 (CI 0.70-0.95).

**Conclusions:**

The annual cumulative incidence of fetal death in our study was 23/1000 live UCP cases delivery per year for the period of 10 years studied. Cesarean section reduced perinatal mortality by a factor of 2. Diagnosis to delivery interval <30 minutes and putting mother in knee chest position were protective against fetal death.

## Introduction

Umbilical cord prolapse (UCP) is a rare obstetric complication usually necessitating emergency delivery and is associated with high perinatal morbidity and mortality.

Reported prevalence of cord prolapse varies between 0.1 and 1.8%. [1-3]. Studies done in United states of America, Thailand, and Israel reported perinatal mortality rate following UCP between 3-15% [4-7]. There has been remarkable improvement from figures as high as 37.5% in the early 1900, probably due to improvements in neonatal care and liberal use of cesarean section on diagnosis [4]. However, perinatal mortality following UCP in some Africa countries is reported as high as 36% [8].

Umbilical cord prolapsed may be overt or occult. In the overt type, there is descent of the cord into the vagina or up to the vulva. Occult UCP is when the cord is alongside the presenting part and is palpable only by passing the examining finger into the cervical canal. Immediate delivery by the fastest feasible and safe means is recommended in UCP to save the baby [6,7]. In the interval before delivery, various interventions are used to try to improve fetal outcomes. These include putting mother in knee-chest position, instillation of fluid into the bladder, giving tocolytics to mothers in labor with UCP and intrauterine resuscitation [9,10].

## Material and methods

This was a retrospective study that covered a period of 10 years. The files of mothers who had UCP and delivered between 1^st^ January 2000 to 31^st^ December 2009 were retrieved from the records department. They were identified by first checking the in-patient numbers of all deliveries which had cord prolapse in Mulago Hospital 5^th^ floor labor suite delivery and theatre operation books. We extracted all the files of mothers who delivered in the general labor ward between 1^st^ January 2000 to 31^st^ December 2009, with pregnancies ≥28 weeks of gestation and live fetuses at the time of diagnosis of UCP.

The maternal and fetal data were reviewed for variables of interest. These included maternal characteristics like: maternal age, occupation, distance from Mulago hospital, marital status, booking status and parity. Fetal characteristics like: whether pregnancy was singleton or multiple, fetal heart pattern, Apgar scores at one and five minutes, birth weight and cord position. Obstetric variables included interventions like knee chest position, cervical dilatation at time of UCP diagnosis, diagnosis to delivery interval and mode of delivery. The information was entered into an extraction form. We excluded files in which there was inadequate data like those missing information on time of diagnosis of cord prolapse, time of delivery of the baby and fetal outcome.

We used STATA 10 for analysis of data.

Incidence of fetal demise was determined by dividing the total number of fetal death following UCP by total number of cord prolapse per year studied for the period of 10 years. Chi-square test was used to show the association between the explanatory variables and fetal outcome. Level of significance was set at 0.05. We fit log binomial models yielding risk ratios and 95% confidence interval, to establish the strength of association between the explanatory variables and fetal death.

### Ethical considerations

The study was approved by the Makerere University College of Health Sciences Research and Ethics Committee, and the Uganda National Council of Science and Technology.

## Results

From the labor ward delivery books and theatre operation books for the 10 year period 2000-2009, we identified 1081 cases of umbilical cord prolapse. However; we were able to retrieve 934 files of which 273 files were excluded because their babies were already dead at the time of diagnosis of UCP and others had inadequate data. Hence 661 files were included in the sampling frame from which 438 files were selected by simple random sampling.

Of the 438 UCP with live fetuses at diagnosis, 101(23%) lost their babies within 24 hours after birth or were delivered dead. This gave annual cumulative incidence of fetal death following UCP of 23/1000 live UCP cases delivered/year.

The mean diagnosis to delivery time was 44 minutes, SD 41.25, (range 4 to 255 minutes). The mean APGAR score at 5 minutes was 6.9., SD 3.88. The mean birth weight was 3.1 kg, SD 0.64 (range 1 to 5 kg).

Social demographic of the women and association with fetal outcome are summarized in Table 1.

**Table 1:** Social demographic characteristics and association with fetal outcome in mothers diagnosed with umbilical cord prolapse in Mulago hospital from year 2000 to 2009

**Variable**	**N**	**(%)**	**Fetal survival n (%)**	**Fetal death n (%)**	**p-value**
**Age**					
<20	92	(21)	70 (76.1)	22 (23.9)	0.626
20-39	343	(78.3)	264 (77)	79 (23)	
≥40	3	(0.7)	3 (100)	0	
**Total**	**438**		**337**	**101**	
**Marital status**					
Married	420	(95.9)	324 (77.1)	96 (22.9)	0.627
Single	18	(4.1)	13 (83.3)	5 (16.7)	
**Total**	**438**		**337**	**101**	
**Work**					
Housewife	388	(88.6)	300 (77.3)	88 (22.7)	
Civil servant	18	(4.1)	15 (83.3)	3 (16.7)	0.527
Others	31	(7.1)	21 (67.7)	10 (32.3)	
**Total**	**437**		**336**	**101**	
**Distance**					
<10 km	236	(53.9)	188 (79.7)	48 (20.3)	0.144
≥ 10 km	202	(46.1)	149 (73.8)	53 (26.2)	
**Total**	**438**		**337**	**101**	

P- Values for chi- square test.

Referred mothers, irregular fetal heart pattern at admission, cord outside vagina, cervical dilation ≥7 cm, mother not put in knee chest position, DDI more than 60 minutes and vaginal delivery were significantly associated with fetal death (see Table 2):

**Table 2:** Obstetric and neonatal characteristics and association with fetal outcome

**Variable**	**N**	**(%)**	**Perinatal survival n (%)**	**Perinatal death n (%)**	**(p-value)**
**Parity**					
1	128	(29.1)	98 (76.6)	30 (23.4)	0.617
2-5	259	(59.1)	197 (76)	62 (24)	
≥6	51	(11.6)	42 (82.4)	9 (17.6)	
**Referral status**					
Referred	49	(11.2)	23 (46.9)	26 (53.1)	0.000
Not referred	389	(88.8)	314 (80.7)	75 (19.3)	
**Booking status**					
Mulago ANC	174	(39.7)	142 (81.6)	32 (18.4)	0.122
Elsewhere	60	(13.7)	42 (70)	18 (30)	
Unbooked	204	(46.6)	153 (75)	51 (25)	
**Fetal heart (N = 437)**					
Regular	297	(67.8)	274 (92.3)	23 (7.7)	0.000
Irregular	140	(32.2)	62 (44.3)	78 (55.7)	
**Cord position (N = 436)**					
Outside vagina	91	(20.8)	47 (51.6)	44 (48.4)	0.000
Inside vagina	334	(76.2)	280 (83.8)	54 (16.2)	
Cervical canal	11	(2.5)	10 (90.6)	1 (9.1)	
**Cervical dilatation**					
≥7 cm	228	(52.1)	160 (70.2)	68 (29.8)	0.000
<7 cm	210	(47.6)	177 (84.3)	33 (15.7)	
**Knee chest position**					
Put	348	(79.5)	279 (80.2)	69 (19.8)	0.002
Not put	90	(200.5)	58 (64.4)	32 (35.6)	
**DDI**					
≤ 30 min	224	(51.1)	197 (87.9)	27 (12.1)	0.000
31-60	115	(26.3)	94 (81.7)	21 (18.3)	
>60	99	(22.6)	46 (46.5)	53 (53.5)	
**Mode of delivery**					
Vaginal	121	(27.6)	79 (65.3)	42 (34.7)	0.000
Caesarean section	317	(72.4)	258 (81.4)	59 (18.6)	
**Maturity**					
Mature	383	(87.4)	295 (77)	88 (23)	0.913
Premature	55	(12.6)	42 (76.4)	13 (23.6)	
**Apgar score at 5 min**					
≥7	305	(69.6)	304 (99.7)	1 (0.3)	0.000
<7	133	(30.4)	33 (24.8)	100 (75.2)	

P- Values for chi square test, DDI = Diagnosis to delivery interval.

On adjusting for confounding, DDI <30 minutes (RR 0.85, 95% CI 0.83-0.88) and putting mother in knee chest position (RR 0 80 95% CI 0.77-0.84) were the two factors protective from fetal demise. The protective effect of caesarean section mode of delivery was negated after controlling for the other variables (see Table 3).

**Table 3:** Multivariate analysis for association with fetal demise

**Factor**	**Adjusted Risk Ratios(RR)**	**95% Confidence interval**
DDI <30min (ref)	0.79	0.74-0.85
Mode of delivery		
(ref=vagina delivery)	1.14	1.02-1.28
Knee chest position (ref)	0.81	0.70-0.95
Cord position (ref=out vagina)	1.03	0.98-1.09
Cervical dilatation (ref=>7 cm)	0.97	0.87-1.09
Referral status (ref=referred)	1.06	0.95-1.18

## Discussion

The overall fetal demise (23%) and annual cumulative incidence of fetal death in pregnancies complicated by UCP in New Mulago hospital was found to be very high for the 10 years period studied. Compared to other studies [5,8] which reported perinatal mortality of 14.2% and 8.3% respectively, the higher perinatal mortality in our study could be attributed to failure to deliver these mothers urgently by emergency caesarean section or vaginally if appropriate once the diagnosis of UCP is made. In more than 70% of the mothers DDI was greater than 30 minutes. Mulago hospital labor ward is very busy and the backlog of emergencies is ever big with many of them life threatening to the mothers. It is possible that these become priorities over cases of UCP. Inadequate theatre facilities and inadequate manpower contribute to failure to reduce on DDI leading to fetal loss.

Some interventional strategies employed by other centres in the developed world are not performed in Mulago due to logistical problems common in low resource settings. These include instillation of fluid into the bladder, giving tocolytics to mothers in labor with UCP and intrauterine resuscitation [9].

In our study, putting mothers in knee chest position was found to be protective against fetal death. The maneuver aims at reducing pressure of the presenting part on the prolapsed cord which may cause partial or complete occlusion. A similar association was demonstrated by a study [10] which reported 1.5% fetal mortality. The higher mortality of 19.8% we found could be due to a larger number of women who had DDI > 30 minutes. The position is uncomfortable for the mother if the diagnosis to delivery interval is prolonged change of position is likely to happen if prolonged.

Like in other studies [6], we found that diagnosis delivery interval (DDI) < 30 minutes was associated with better fetal survival.

Although DDI is used as a measure of obstetric management efficiency, our results showed mortality of 12.1% for the babies delivered within 30 minute which was still high. This might be due to inadequate resuscitation facilities in our crowded hospital. On the other hand delayed diagnosis of the problem may imply that the fetus has been compromised longer by hypoxia. Otherwise it is obvious that the earlier delivery is conducted after diagnosis, the better the fetal outcome.

We found that vaginal delivery was significantly associated with perinatal death and caesarean section reduced perinatal mortality by a factor of 2. Other studies reported even better fetal outcomes [6,11,12]. The higher cesarean section rates found inpregnancies with this complication are thus justifiable.

We found that a cord found outside the vagina was associated with more deaths compared with cord palpated inside the vaginal canal. Cooler temperatures outside the vagina and exposure of the prolapsed cord to the external environment results into vasospasms of umbilical cord vessels leading to fetal hypoxia. Other studies reported similar findings [13,14].

The findings of our study were generally not new. Most studies in the literature have similar findings but most report lower perinatal mortality. This could be due to the sheer numbers of deliveries in Mulago hospital, which currently average about 20 every 24 hours. Inadequate theatre facilities and understaffing lead to longer DDI. While reducing DDI is the surest way to reduce fetal mortality, further research and investment in intrauterine resuscitation techniques as delivery is awaited would be worthwhile.

## Conclusion

The incidence of fetal demise following umbilical cord prolapsed at Mulago hospital is very high.

Putting mothers with UCP in knee chest position helps reduce fetal demise but the effect is lost if delivery is not conducted in the shortest possible time. There is urgent need to invest in theatre facilities and manpower to achieve this.

## Abbreviations

UCP: Umbilical cord prolapsed; DDI: Diagnosis to delivery interval; ANC: Antenatal care; RR: Risk ratio; CI: Confidence interval; IP: In-patients number.

## Competing interests

The authors declare that they have no competing interests.

## Authors’ contributions

EWW, SN and TM participated in development of the concept and full proposal, plus seeking approval from ethical committees. EWW did the data collection, entry and cleaning. TM and EWW performed the analysis. All authors participated in the interpretation of results and writing of the manuscript. All authors read and approved the final manuscript.
